# Sex-dependent temporal changes in astrocyte-vessel interactions following diffuse traumatic brain injury in rats

**DOI:** 10.3389/fphys.2024.1469073

**Published:** 2024-09-25

**Authors:** Zackary Sabetta, Gokul Krishna, Tala Curry-Koski, Mackenzie Lopez, P. David Adelson, Theresa Currier Thomas

**Affiliations:** ^1^ Department of Child Health, University of Arizona College of Medicine-Phoenix, Phoenix, AZ, United States; ^2^ A.T. Still University Kirksville College of Osteopathic Medicine, Kirksville, MO, United States; ^3^ Translational Neurotrauma and Neurochemistry, Barrow Neurological Institute at Phoenix Children’s Hospital, Phoenix, AZ, United States; ^4^ West Virginia University School of Medicine, Rockefeller Neuroscience Institute, Morgantown, WV, United States; ^5^ Phoenix VA Healthcare System, Phoenix, AZ, United States

**Keywords:** diffuse axonal injury, cerebrovascular astrocytes, blood-brain barrier, glial fibrillary acidic protein, aging with TBI, chronic neurodegeneration, perivascular astrocytes

## Abstract

Traumatic brain injury (TBI) is associated with diffuse axonal injury (DAI), a primary pathology linked to progressive neurodegeneration and neuroinflammation, including chronic astrogliosis, which influences long-term post-TBI recovery and morbidity. Sex-based differences in blood-brain barrier (BBB) permeability increases the risk of accelerated brain aging and early-onset neurodegeneration. However, few studies have evaluated chronic time course of astrocytic responses around cerebrovascular in the context of aging after TBI and sex dependence. We observed increased glial fibrillary acidic protein (GFAP)-labeled accessory processes branching near and connecting with GFAP-ensheathed cortical vessels, suggesting a critical nuance in astrocyte-vessel interactions after TBI. To quantify this observation, male and female Sprague Dawley rats (∼3 months old, n = 5–6/group) underwent either sham surgery or midline fluid percussion injury. Using immunohistochemical analysis, we quantified GFAP-labeled astrocyte primary and accessory processes that contacted GFAP-ensheathed vessels in the somatosensory barrel cortex at 7, 56, and 168 days post-injury (DPI). TBI significantly increased GFAP-positive primary processes at 7 DPI (*P* < 0.01) in both sexes. At 56 DPI, these vessel-process interactions remained significantly increased exclusively in males (*P* < 0.05). At 168 DPI, both sexes showed a significant reduction in vessel-process interactions compared to 7 DPI (*P* < 0.05); however, a modest but significant injury effect reemerged in females (*P* < 0.05). A similar sex-dependent pattern in the number of accessory processes provides novel evidence of long-term temporal changes in astrocyte-vessel interactions. TBI-induced changes in astrocyte-vessel interactions may indicate chronic BBB vulnerability and processes responsible for early onset vascular and neurodegenerative pathology.

## 1 Introduction

Traumatic brain injury (TBI) presents one of the most complex neurological insults. Each year, millions of individuals suffer from TBI, raising significant concern about the long-term health effects (Mayer et al., 2017). Chronic consequences of TBI accelerate brain aging, where predicted brain age differences were 5 years greater than controls (Dennis et al., 2024). TBI is also associated with an increased long-term risk of early-onset cardiovascular diseases, stroke, dementia, and neurodegenerative diseases (Kiraly and Kiraly, 2007; Stewart et al., 2022; Schneider et al., 2023). Diffuse axonal injury (DAI) is one of the most common and potentially most insidious pathological features due to its involvement in chronic pathogenesis, where the magnitude of DAI links with symptom severity in clinical and preclinical studies (Johnson et al., 2013; Smith et al., 2013; Blennow et al., 2016). Traumatic DAI initiates chronic neurodegeneration, neuroinflammation, and oxidative stress, leading to chronic blood-brain barrier (BBB) dysfunction and maladaptive vascular remodeling, all of which are implicated in accelerating brain aging (Faden and Loane, 2015; Cash and Theus, 2020; Lin et al., 2022; Lu et al., 2023). These TBI-induced changes accelerate brain aging, resulting in cerebrovascular impairment, an early indicator of cognitive decline and a hallmark of age-related neurodegenerative diseases (Senatorov et al., 2019; Barisano et al., 2022; Knox et al., 2022; Sulimai et al., 2023).

Astrocytes are major glial cells that establish direct structural and functional contact with vasculature to regulate BBB permeability, blood flow, energy uptake, and waste clearance (Koehler et al., 2009; Mathiisen et al., 2010; Baldwin et al., 2023). Astrocyte endfeet directly interact with the cerebral vessels, particularly endothelial cells, the basement membrane, and pericytes, and are critical for the formation and maintenance of the BBB (Cabezas et al., 2014). In pathological conditions, astrocyte reactivity increases the expression of glial fibrillary acidic protein (GFAP), which is associated with hypertrophy, proliferation, and changes in function, making it a standard and widely utilized marker for studying the response to TBI and the influence of interventions. It is important to note that astrocyte pathophysiology is an emerging field with a growing number of phenotypes and classifications (Verkhratsky et al., 2023). This manuscript will use the term “reactive astrocytes” as a generalized descriptor for increased GFAP-expressing astrocytes. Chronic astrocyte reactivity after TBI, reported in clinical and preclinical studies, presents a diversity of phenotypes that can contribute directly to vascular pathology (George et al., 2022). Astrocyte endfeet ensheath the vasculature, and disruptions to this dynamic interaction affect global neurological function (Mills et al., 2022). Astrocyte reactivity after TBI results in morphological and functional changes that may be either beneficial (supporting homeostasis and BBB repair) or detrimental (promoting BBB permeability and impaired blood flow) (Burda et al., 2016; Munoz-Ballester and Robel, 2023). Chronic astrocytic reactivity is a key player in pathology progression, contributing to vascular damage and the development of inflammatory cascades (Kempuraj et al., 2021). Morphological remodeling of reactive astrocytes leads to loss of gliovascular interaction, which can influence the barrier properties after TBI (Villapol et al., 2014). Additionally, vascular aging disrupts astrocyte associations with blood vessels, further increasing barrier permeability (Dunn et al., 2021). These processes are implicated in long-term recovery and morbidity, significantly affecting BBB permeability and increasing the risk of accelerated brain aging and neurodegenerative diseases.

Sex differences in healthy and disease-associated cerebrovascular aging, astrocyte reactivity, and vascular cognitive impairment have been well-documented in the literature (Robison et al., 2019). TBI accelerates brain aging partly due to BBB dysfunction, with astrocytes playing a crucial role in cerebrovascular pathology. Notable sex differences exist in astrocytic responses to TBI, yet astrocyte-vascular interactions and how they differ by sex over time have not been fully evaluated, highlighting the importance of sex-specific research to address distinct impacts on vascular health and neurodegenerative risks (Chisholm and Sohrabji, 2016; Honarpisheh and McCullough, 2019; Hubbard et al., 2022; Cantone et al., 2023; Zhang et al., 2023). This gap underscores the need to evaluate astrocytic responses over time, particularly their interactions with blood vessels, as several studies have shown their implications in both the short- and long-term effects of TBI (Mira et al., 2021). However, the findings are often inconsistent due to variations in injury types, evaluation time points, inclusion of female subjects, and outcome measures (Burda et al., 2016; Munoz-Ballester and Robel, 2023; Verkhratsky et al., 2023). Most existing profiles are limited to evaluations under 2 months post-injury, focus predominantly on males, and often involve penetrating injuries associated with glial scar formation (Villapol et al., 2014). The use of diverse outcome measures and differences in the duration of astrocyte reactivity and molecular profiles add to the complexity, making interpretation across different preclinical models and time points challenging. To date, few studies have extended temporal assessments of astrocyte-vessel interactions to 6 months post-injury with sex as a biological variable using a highly reproducible DAI model without cavitation. The few supporting reports confirm unique astrocyte phenotypes associated with chronic BBB dysfunction (George et al., 2022). Temporal profiles of astrocyte-vessel interactions after DAI are needed to address these gaps for a comprehensive understanding of astrocytic roles in TBI and their contributions to accelerated brain aging (Badaut and Bix, 2014; Díaz-Castro et al., 2023).

**FIGURE 1:**
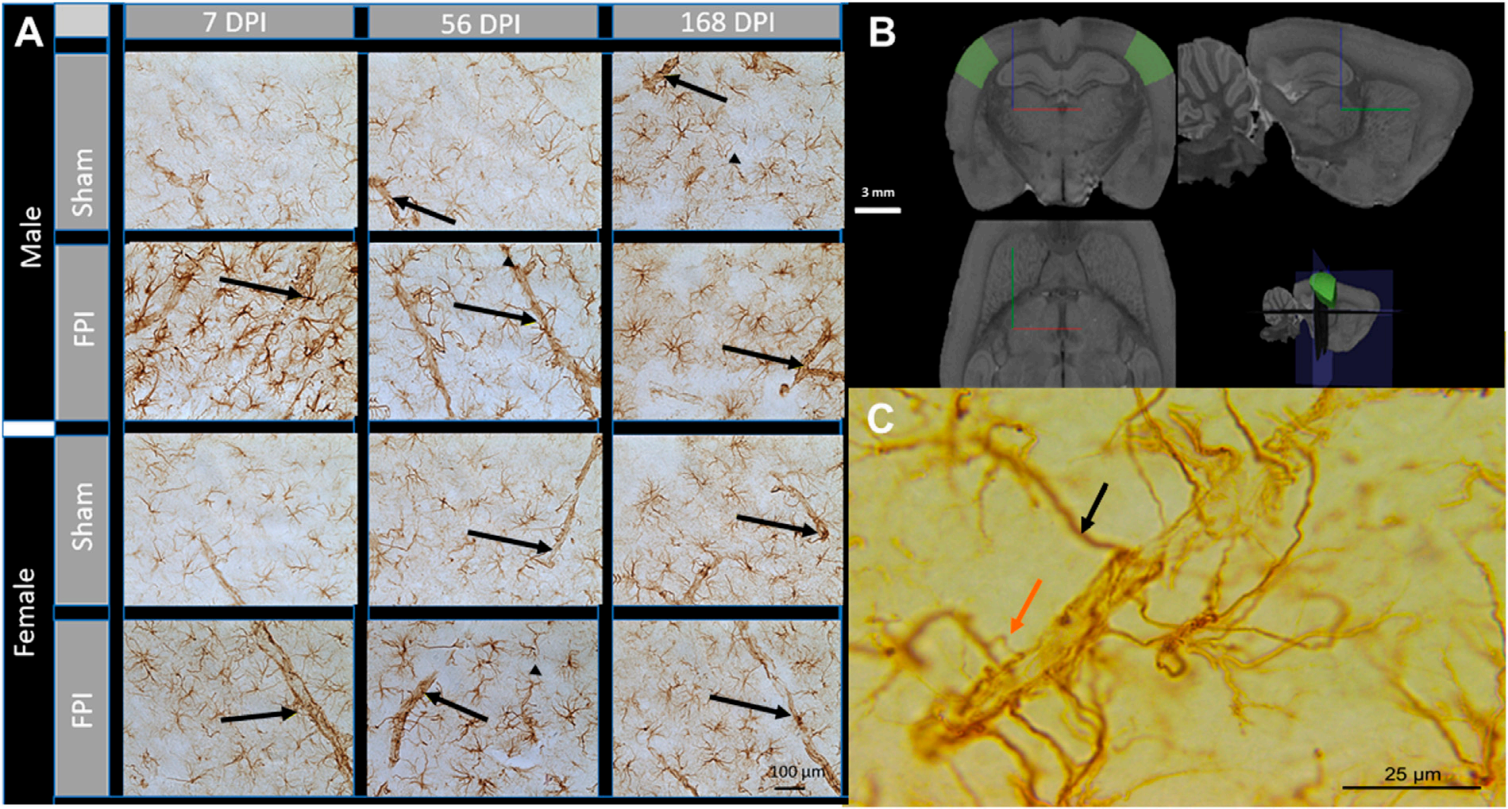
Astrocyte interaction with blood vessels after TBI. **(A)** Representative images of GFAP immunohistochemistry in the S1BF acquired at 40× magnification. Black arrows indicate GFAP-ensheathed vasculature. At 7 DPI, astrocytes displayed reactive morphology with an increased number of cells, larger cell bodies, and more pronounced processes. Scale bar = 100 **(B)** 3D schematic showing the S1BF, where images of vessels were captured within cortical layer IV (green areas). Brain region defined by the Waxholm Space Atlas of the Sprague Dawley rat brain (v4) (Papp et al., 2014). White scale bar = 3 mm; red, blue, and green lines represent x, y, and z directions, respectively. **(C)** A scaled-up image of GFAP + processes ensheathing a cortical vessel. The black arrow indicates a primary process directly from the astrocyte, and the orange arrow indicates an accessory process branching proximal to GFAP + ensheathment. Scale Bar = 25 µm.

We previously demonstrated that GFAP density was significantly increased in the primary somatosensory cortex (S1BF; Figure 1B) at 7- and 56-days post-injury (DPI) and that GFAP intensity increased in shams over 6 months post-surgery (Sabetta et al., 2023). GFAP positive (+) astrocyte processes ensheathed vessels were clearly distinguished by their larger diameter and perpendicular or lateral trajectory to the brain surface, cylindrical shape, and clear lumen, with apparent changes in astrocyte-vessel interactions (Figure 1A). At 100× magnification, we predominantly observed GFAP-primary processes with no obvious process branches proximal to the GFAP ensheathed vessels (black arrow Figure 1C). At a subacute time point post-injury, we detected an increased number of *accessory processes* branching proximal to the vessels and contacting the GFAP + ensheathment around the vessel (orange arrow in Figure 1C), indicating a novel nuance in astrocyte-vessel interactions. We sought to determine the changes in primary and accessory GFAP + processes that interact with vessels to assess if a single TBI chronically disrupts astrocyte-vessel interactions indicative of chronic BBB vulnerability in male and female rats at 7-, 56-, and 168-days post-injury in a rat model of diffuse TBI.

## 2 Methods

### 2.1 Animals

A total of 64 young adult age-matched male and naturally cycling female Sprague-Dawley rats (3–4 months old; males 367 ± 3 g and females 235 ± 1.5 g; n = 5–6/group; Inotiv (formerly Envigo), Indianapolis, IN, United States of America) from the same study were used in these experiments. Rats were housed in temperature (68°F–79°F) and humidity-regulated 12:12 h light:dark cycle room with free access to food (Teklad 2918) and water (Innovive, San Diego, CA, United States of America) and acclimatized for at least 1 week before experiments. All procedures were conducted in compliance with ARRIVE guidelines and consistent with the National Institutes of Health (NIH) Guidelines for the Care and Use of Laboratory Animals approved by the Institutional Animal Care and Use Committee (protocol #18-384) at the University of Arizona College of Medicine-Phoenix.

### 2.2 Surgeries

Midline fluid percussion injury (FPI) induces DAI without cavitation or contusion (Lifshitz et al., 2016). The surgery was performed similarly to our previous publications (Bromberg et al., 2020; Krishna et al., 2020; Sabetta et al., 2023). Cages of rats (2/cage) were randomized to either FPI or sham groups. Briefly, rats were anesthetized with isoflurane (5% in 100% oxygen for 5 min) prepared for aseptic surgery and placed into a stereotaxic frame (Kopf Instruments, Tujunga, CA) and anesthesia maintained at 2.5% for the procedure’s duration. A 4.8 mm circular craniectomy was centered on the sagittal suture midway between bregma and lambda, and the skull flap was removed carefully, ensuring the underlying dura and superior sagittal sinus remained intact. An injury hub was fixed directly over the craniectomy using cyanoacrylate gel and methyl-methacrylate (Hygenic Corp., Akron, OH) and filled with 0.9% sterile saline. The incision was then partially sutured closed on the anterior and posterior edges with 4.0 Ethilon sutures. Topical lidocaine and antibiotic ointment were applied. Rats were returned to a pre-warmed holding cage post-surgery and monitored until ambulatory.

### 2.3 Midline FPI

Two hours following surgical procedures and the return of ambulation, rats were re-anesthetized, the hub was filled with 0.9% sterile saline, and attached to the male end of a fluid percussion device (Custom Design and Fabrication, Richmond, VA). After the return of a pedal withdrawal response, an injury averaging 1.8–2.0 atmospheric pressure (atm) for males and 1.7–1.9 atm for females was administered by releasing the pendulum (from 16° for males and 15.5° for females) onto the fluid-filled cylinder. Shams were attached to the device, but the pendulum was not released after a positive pedal withdrawal response. Immediately after administration of the injury, the fencing response, apnea, seizures, and the return of righting reflex were recorded for brain-injured animals, and the hub was removed *en bloc* (McIntosh et al., 1987; Hosseini and Lifshitz, 2009). Inclusion criteria required that injured rats have a righting reflex time ranging from 6 to 10 min and a fencing response indicative of mild-to-moderate TBI. Rats were re-anesthetized, the surgery site inspected for herniation and dural integrity, the incision was closed, and topical lidocaine and antibiotic ointment were applied. Rats were then placed in a pre-warmed holding cage for recovery. Post-operative monitoring was performed for 5 days by physical evaluation. Rats were pair-housed in the same room according to injury status and sex throughout the study. All cohorts were time post-injury matched, with age- and sex-matched shams at each time point.

### 2.4 Histology

Brains were collected at 7-, 56-, and 168 days post-injury (DPI), rinsed with ice-cold phosphate-buffered saline (PBS), hemisected, and one hemisphere post-fixed in 4% paraformaldehyde for 24 h, transferred to fresh PBS with sodium azide, and shipped to Neuroscience Associates Inc. (Knoxville, TN). Brains were coded and randomized into two gelatin blocks (MultiBrain^®^ Technology, NeuroScience Associates, Knoxville, TN) to be batch-processed for simultaneous histological and immunohistochemical staining. 40 μm thick sections were taken in the coronal plane, stained with glial fibrillary acidic protein (GFAP); primary Ab: Dako, Z0334, 1:75,000; secondary Ab: Vector, BA-1000) using the free-floating technique and visualized using 3,3′-Diaminobenzidine (DAB).

### 2.5 Imaging and analysis

Image capture and analysis was completed by investigators blinded to injury status, days post-injury, and sex. Images were acquired on an upright Zeiss Axio Imager 2 equipped with a Hamamatsu ORCA-flash 4.0 digital camera (catalog #C13440). An oil-immersion 100×/1.2 C-Apochromat lens was used to capture one image in the S1BF localized to layer 4 per 3 adjacent sections per animal using Paxinos and Watson atlas (Figure 1B) (Paxinos and Watson, 2007). A total of 192 images were taken for GFAP-positive stained astrocytes connected to the GFAP-ensheathed cerebral vasculature using Neurolucida software (MBF) and exported to ImageJ (National Institutes of Health) software. The number of primary astrocyte processes connecting to GFAP-ensheathed vessels and accessory branches near the vessels were quantified to assess astrocyte contributions to the BBB (Figure 1C). Vessel length was measured using the line tool in ImageJ software scaled in micrometers to normalize process counts to the vessel length as a quantitative metric for comparison (Abramoff et al., 2004). The average vessel length was similar between all groups, with an overall average of 132.5 ± 1.54 µm. Measurements from the 3 adjacent sections were averaged (per rat) for statistical analysis. Image analysis was carried out by two investigators. Data were analyzed to address the following questions for each sex: (1) Effect of FPI: Was there an impact of FPI? (2) Effect of time post-FPI: Did the timing post-FPI indicate aging with injury, and if so, when? (3) Effect in shams over time: Were there changes in sham groups over time (3 months at time of injury, 9 months at 168 DPI)? If so, when? (4) Sex-related effects or interactions: Was there evidence of any potential effects or interactions related to sex?

### 2.6 Statistics

Group sizes were determined from previous publications, primarily using male rats at 28 DPI (Hoffman et al., 2017; Thomas et al., 2018; Beitchman et al., 2019; Bromberg et al., 2020), where an n = 5 per group was shown to provide >90% power to detect a significant increase in GFAP intensity after FPI with a representative effect size of *d* = 3.4 (Thomas et al., 2018). However, due to the exploratory nature of this study, including chronic time points and the inclusion of females, data were analyzed separately for each sex to ensure appropriate power. A two-way ANOVA was initially conducted with factors of injury (FPI vs. sham) and days post-injury (DPI; 7 vs. 56 vs. 168). To explore potential sex differences, a subsequent three-way ANOVA was performed with sex (male vs. female) as an additional factor. If significant effects or interactions involving sex were identified (at *P* < 0.05), they are reported in the results section. Normality was assessed using the Kolmogorov-Smirnov test, and homogeneity of variances was evaluated using the Brown-Forsythe test. When these assumptions were violated, data were log-transformed to meet the criteria. Reported statistics include results from transformed data, though raw data are presented in all graphs for clarity. Tukey’s *post hoc* tests were performed to clarify significant main effects and interactions identified by ANOVA, (*P* < 0.05). Data are presented as mean + SEM. Outliers were identified using ROUT analysis (Q = 1%), and 1 outlier was detected (male 7 days sham). Shams were compared across MultiBrain^®^ blocks and time points to detect potential block or cohort effects, and no significant differences were detected. All statistical analyses were performed using GraphPad Prism (version 10.2.2).

## 3 Results

### 3.1 DAI caused an increased number of GFAP-positive primary processes per vessel length at 7 DPI with sex-dependent long-term trajectories

**FIGURE 2:**
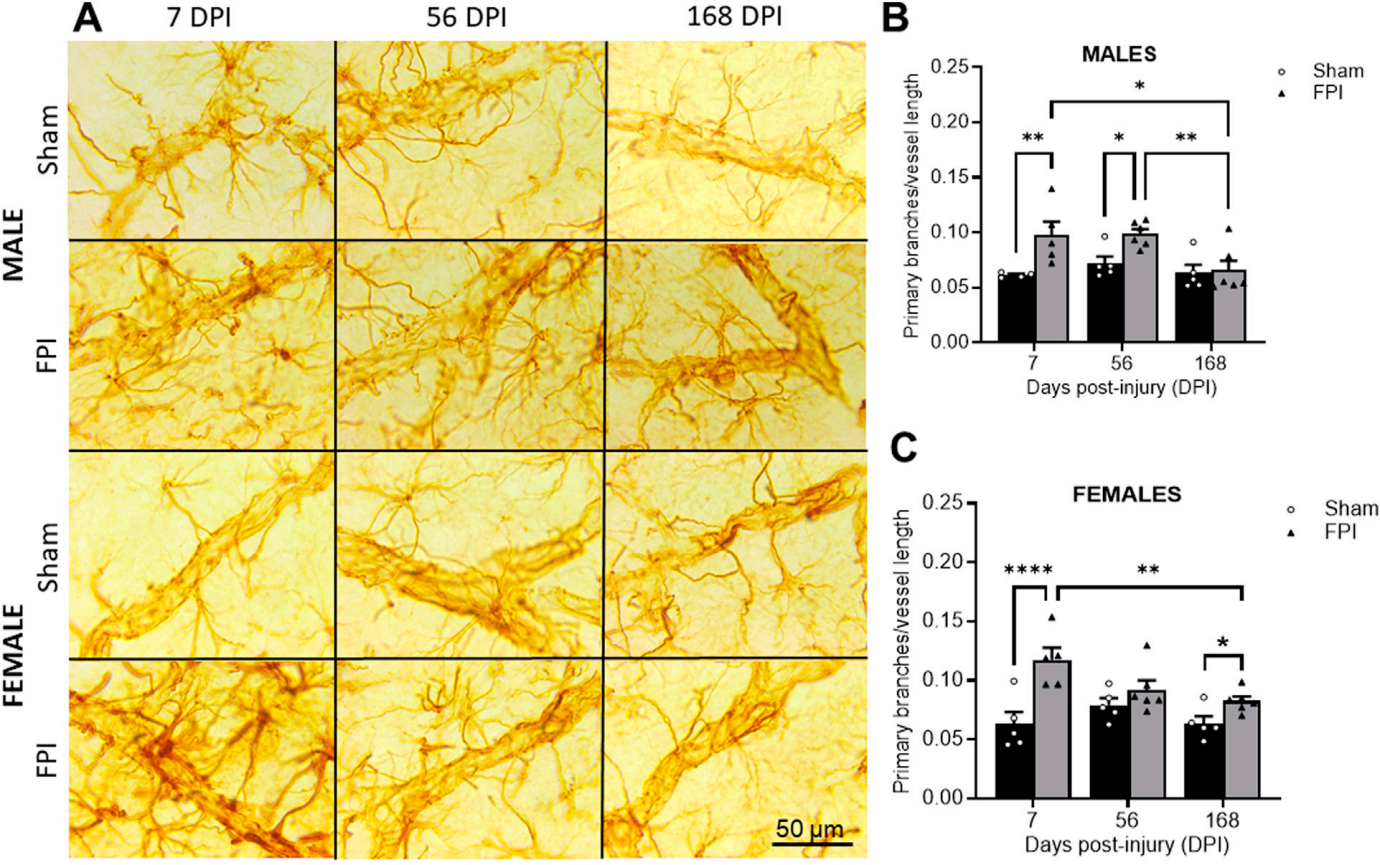
FPI caused an increased number of GFAP + primary processes per vessel length at 7 DPI that remained elevated longer in males. **(A)** Representative 100× GFAP immunostained images within layer IV of the S1BF region of sham and FPI rats at 7, 56, and 168 DPI of both sexes. **(B)** In males, an injury effect was detected at 7 and 56 DPI compared to age- and sex-matched shams. The number of processes decreased in FPI rats to sham levels by 168 days. **(C)** In females, FPI increased primary processes at 7 DPI compared to sham. Process numbers significantly declined between 7 and 168 DPI, where an effect of FPI was still present at 168 DPI *N*=5–6/sex. Data were analyzed by two-way ANOVA followed by Tukey’s multiple comparison tests. *P < 0.05, **P < 0.01, and ****P < 0.0001. Error bars indicate + SEM. Scale bar = 50 µm.

Representative 100× photomicrographs of GFAP-positive processes ensheath vessels in the S1BF (Figure 2A). As shown in Figure 2B, two-way ANOVA revealed a significant effect of FPI (F_1,25_ = 11.87, *P* = 0.002) and DPI (F_2,25_ = 5.34, *P* = 0.012) in males. Post-hoc analysis reveals significant differences between FPI and sham at 7 DPI (*P* < 0.01) and 56 DPI (*P* < 0.05), with the number of processes in FPI animals returning to sham levels by 168 DPI. In females, primary process numbers varied as a function of FPI (F_1, 26_ = 23.96, *P* < 0.0001) and FPI × DPI interaction (F_2, 26_ = 4.16, *P* = 0.027). The follow-up comparisons indicated that FPI increased the primary processes at 7 DPI compared to shams (*P* < 0.0001). While processes significantly decreased between 7 DPI and 168 DPI (*P* < 0.05), a small but significant effect was present between sham and FPI at 168 DPI (Figure 2C). We measured no changes in sham rats as a function of time. A three-way ANOVA indicated no sex differences or sex interactions.

### 3.2 FPI increases in GFAP-positive accessory processes parallel FPI-induced increases in primary processes

**FIGURE 3:**
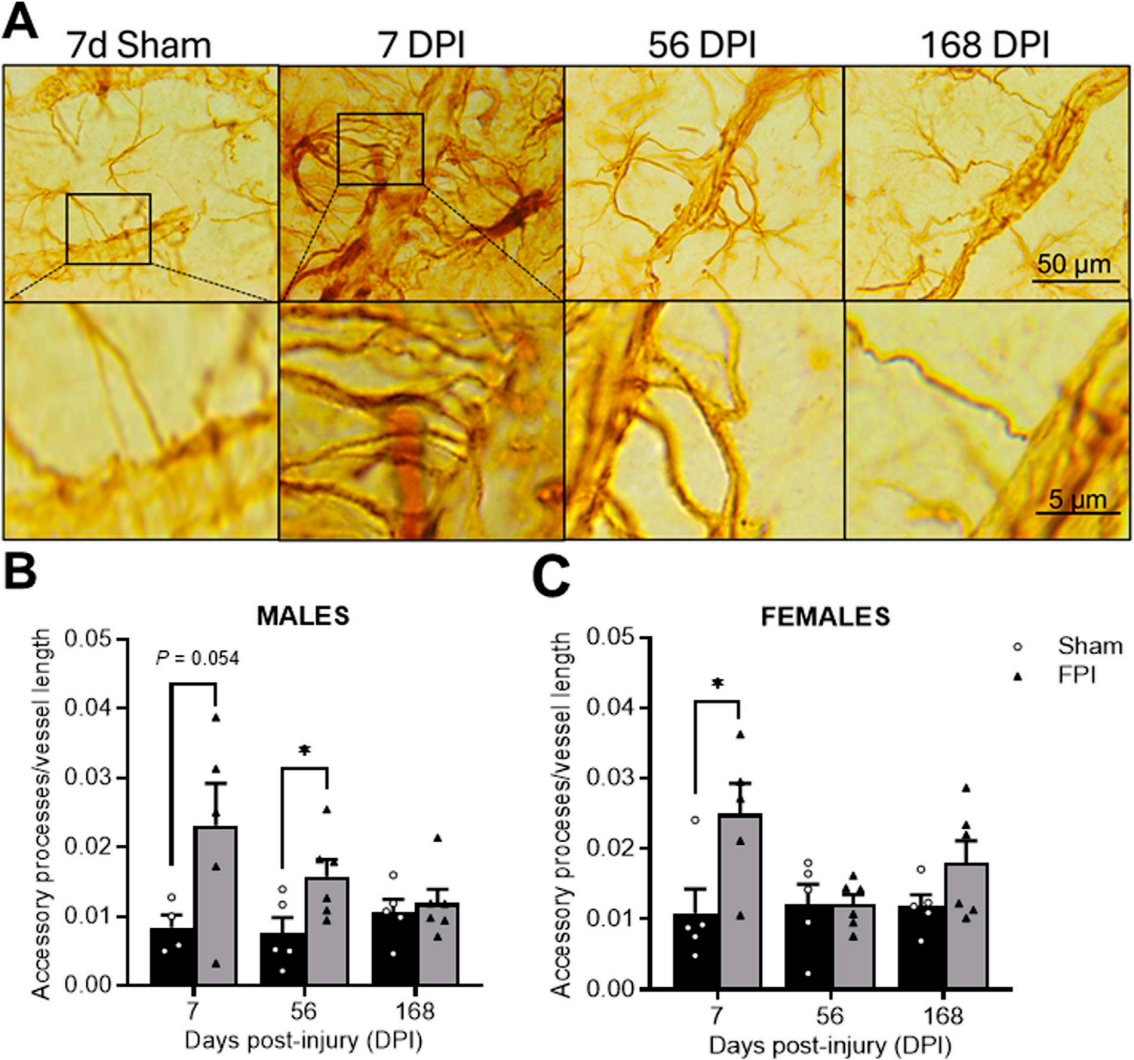
TBI promotes time-dependent changes in the number of GFAP-labeled accessory processes. **(A)** Representative GFAP immunostained images within the S1BF region of sham and FPI rats at 7, 56, and 168 DPI from both sexes to highlight changes in accessory processes over time. The top row are images captured at 100×. The bottom row images magnify the accessory process interactions with the vessel. **(B)** In males, an FPI increased accessory processes at 56 DPI compared to age- and sex-matched shams. **(C)** In females, FPI increased primary processes at 7 DPI compared to sham *N*=5–6/sex. Data were analyzed by a two-way ANOVA followed by Tukey’s multiple comparison tests. *P < 0.05 and **P < 0.01. Error bars indicate + SEM. Scale bar = 50 µm.

Representative photomicrographs of GFAP-positive accessory processes ending contributing to GFAP-ensheathed vessels in the S1BF (Figure 3A). In males, an effect of FPI was measured (F_1, 25_ = 7.76; *P* = 0.010), where a *post hoc* analysis identified significance between FPI and sham at 56 DPI (*P* < 0.05; see Figure 3B). As shown in Figure 3C, in females, an effect of FPI was also measured (F_1, 26_ = 6.95; *P* = 0.014), where a *post hoc* analysis identified significance between FPI and sham at 7 DPI (*P* < 0.05). Sham rats did not change as a function of time. A three-way ANOVA indicated no sex differences or interactions.

## 4 Discussion

We sought to evaluate temporal changes in astrocytes interacting with the vasculature as a function of time post-injury and sex. Our results demonstrate chronically increased number of primary processes in contact with the GFAP + ensheathment around blood vessels with proximal accessory branches tending to co-occur at 7 DPI (both sexes), 56 DPI (in males), and 168 DPI (in females). Despite measuring overall GFAP reactivity increased in sham controls (Sabetta et al., 2023), the increase in proximal accessory branches was unique to brain-injured rats. While temporal profiles of the sexes have subtle differences, *a posteriori* three-way ANOVAs did not detect significant effects of sex.

The S1BF was strategically chosen for this study due to its large anatomical representation of the whisker barrel circuit, along with dense and well-organized vascular network and distinct cortical layers, offering greater reproducibility in measuring interactions across animals. Larger diameter cortical vessels with a perpendicular or lateral trajectory are characteristics of intracortical arterioles that are the gateway of blood supply to the deeper brain structures. Cortical arterioles are known to be compromised during aging and neurodegenerative diseases, playing a critical role in maintaining cerebral perfusion, with their dysfunction linked to cognitive decline and dementia (Kress et al., 2014). Post-TBI vascular dysfunction may be more prominent at 6 months post-injury in the cortex, where fluid percussion injury induces spreading depolarization, acute hypoperfusion, and changes in neurovascular volume (Ziebell et al., 2016; Balanca et al., 2017). Astrocytes in the S1BF show increased GFAP immunodensity up to 2 months post-injury, where the vast majority (>90%) are in direct vascular contact (Hösli et al., 2022; Sabetta et al., 2023), potentially contributing to vascular dysfunction. In this DAI model, the somatosensory cortex is known for its highly reproducible late-onset and persistent hypersensitivity to whisker stimulation, with several known glial, neuronal, and functional changes over time (McNamara et al., 2010; Thomas et al., 2012; Lafrenaye et al., 2014; Thomas et al., 2017; Thomas et al., 2018; Krishna et al., 2020). Together, this information can be expanded in future studies to comprehensively evaluate the chronic impact of altered astrocyte-vessel interactions within behaviorally relevant nuclei and white matter tracts, focusing on how these changes affect vascular and circuit function in the development of behavioral deficits or recovery post-TBI.

The BBB integrity depends on astrocyte coverage of the vascular surface, with evidence indicating that the alterations in gliovascular interactions are more prominent in neurological disorders and age-related neurodegenerative diseases (Alvarez et al., 2013). Disruptions of astrocyte-vascular interaction could lead to impaired hemodynamic responses and loss of neurovascular coupling, perpetuating pathology progression (Zlokovic, 2011). The primary GFAP-positive processes are major long branches that emanate directly from the soma and continue to envelop vasculature (Khakh and Sofroniew, 2015). Our previous work showed increased GFAP immunoreactivity at 7 and 56 DPI in the sensory cortex, which was similar between males and females after experimental TBI (Sabetta et al., 2023). These data indicate chronic changes in astrocyte vessel interactions with accessory processes following a similar temporal profile as the TBI-induced increase in primary processes, clearly indicating that an increase in accessory processes is injury-mediated and an associated pathology with increased astrocyte-vessel interactions. Considering our previous outcomes, these data indicate that the distribution of GFAP may differ between males and females over the measured times post-injury. Further, this observation may indicate that females have a differentially regulated astrocytic response due to altered protective mechanisms, hormonal regulation, or inflammatory responses. Estrogens can exert a protective action on vascular surfaces by promoting recovery of endothelial cell loss after vascular damage (Krasinski et al., 1997); however, well-powered studies and more detailed examination of alternate outcomes are needed to support this speculation.

Given that astrocytes undergo acute, subacute, and chronic morphological and functional remodeling after TBI, this observation is consistent with previous studies after lateral FPI demonstrating increased arborization in A1 (GFAP+/C3+) astrocytes at 7 DPI (Clark et al., 2019), indicating a pathological astrocyte phenotype; albeit these were ipsilateral to a focal injury. Relatedly, astroglial reactivity with the appearance of longer processes was also observed at 1, 7, and 30 DPI in the somatosensory region of mice; however, these changes were not associated with vascular interactions (Clément et al., 2020). Alternatively, increased astrocyte processes observed at 7 DPI after the diffuse TBI may indicate ongoing adaptive changes to facilitate vascular repair by promoting BBB integrity, providing metabolic support, synaptic activity, and controlling blood flow (Attwell et al., 2010). Further investigation is needed to determine whether these responses are beneficial or detrimental in the case of DAI. Astrocytes extend highly branched processes that split into secondary and tertiary accessory processes, which could be indicative of increased endfeet in contact with blood vessels (Baldwin et al., 2023). Experimental TBI studies have reported time-course changes in cortical vascularization with the appearance of revascularization at 7 DPI (Jullienne et al., 2018), which may explain the increase in astrocyte processes. Aging is known to induce astrocytic gliosis, swelling of endfeet, and loss of interaction with the vasculature, which may be linked to neurodegeneration (Duncombe et al., 2017; Bors et al., 2018). Differences in outcomes may be related to our 6-month post-injury time point being in a relatively young rat (9 months old). Evaluating the effects at 18+ months post-injury could provide a more comprehensive understanding of the chronic consequences of DAI on astrocyte-vessel interactions.

In terms of mechanisms, it was previously reported that acute neurodegeneration may precede reactive astrogliosis, promoting neurovascular reformation (Villapol et al., 2014). Our previous publications indicate ongoing neurodegeneration in both male and female rats, persisting up to at least 2 months in the S1BF and 6 months in deeper nuclei after DAI (Lifshitz and Lisembee, 2012; Thomas et al., 2018; Sabetta et al., 2023). This neurodegeneration can lead to prolonged phagocytosis driven by activated microglia/macrophages, which are in bidirectional communication with endothelial cells and release pro-inflammatory factors (Dudvarski Stankovic et al., 2016). The crosstalk between astrocytes and microglia/macrophages can alter the astrocytic phenotype, influencing astrocyte-vessel interactions that may promote BBB permeability, neurovascular remodeling, neurovascular uncoupling, metabolic alterations, and changes in astrocytic endfeet-enriched proteins (Abbott et al., 2006; Yi and Hazell, 2006; Salehi et al., 2017; Matejuk and Ransohoff, 2020; George et al., 2022). The culmination of these events could create a feedback loop that exacerbates both neurodegeneration and vascular dysfunction and thereby changes in astrocyte morphology. However, it is also important to consider the potential for protective mechanisms, such as the ongoing clearance of debris, release of neurotrophic factors, and induction of anti-inflammatory states, which can promote neuronal survival, tissue repair, regenerative neuroplasticity, and BBB integrity (Simon et al., 2017; Mira et al., 2021). The chronic presentation of accessory processes could indicate enhanced coverage of the BBB and be protective of functional impairment in the vascular microenvironment. The load of neurodegeneration or promotion of adaptive processes may shift the balance between chronic pathophysiology and repair, determining whether the system leans towards exacerbating or mitigating vascular dysfunction (Thomas et al., 2015). Further investigation into the genotype and phenotype of interacting astrocytes may elucidate their functional roles, where these interactions may serve as biomarkers to monitor traumatically induced vascular damage, recovery, and response to interventions, particularly during the subacute and chronic post-injury periods.

With the role of chronic astrocyte reactivity becoming increasingly recognized as a contributor to vascular pathology following TBI, this study is, to the best of our knowledge, the first to highlight novel sub-acute and chronic astrocyte-vessel interactions that may have significant implications for sex-dependent trajectories following diffuse TBI (George et al., 2022; Díaz-Castro et al., 2023). While these findings present intriguing biomarkers for future research, there are limitations to be considered. Results were limited to the evaluation of GFAP + processes, where additional markers could help identify arterioles from venules to indicate if accessory processes are associated with one or the other. Additional markers for endothelial cells, pericytes, or the blood-brain barrier would provide more detail about interactions with specific vascular components. Incorporating markers of glymphatic clearance, hypoxia, and vascular integrity may indicate functional impact. Results are not directly correlated with behavior, however, the somatosensory cortex is the highly integrated cortical relay responsible for the development of the reproducible late-onset and persistent hypersensitivity to whisker stimulation previously reported from our lab and others (McNamara et al., 2010; Thomas et al., 2012; Lafrenaye et al., 2014; Thomas et al., 2017; Thomas et al., 2018; Krishna et al., 2020). Behavioral assessments were intentionally excluded from the experimental design due to indications of chronic HPA axis dysregulation where perceived stressors could potentially influence astrocyte morphology and confound data interpretation (Rowe et al., 2016; Hoffman et al., 2017; Beitchman et al., 2019; Bromberg et al., 2020; Rowe et al., 2020; Fulop et al., 2023; Valenza et al., 2024). Additionally, examining the modulation of ovarian hormones could further elucidate the observed sex-dependent differences.

## 5 Conclusion

Our work provides novel evidence of sex-dependent temporal changes in astrocyte-vessel interactions post-TBI, highlighting significant differences in how males and females respond to brain injury over time. The observed increase in accessory astrocyte processes and their connection with cortical vessels underscores the critical role of astrocytes in maintaining barrier integrity and the potential implications for chronic BBB disruption. These findings suggest that changes in astrocyte morphology may serve as valuable biomarkers for assessing the impact of TBI on vascular function. Understanding nuanced interactions offers a promising avenue for developing targeted treatments, where early interventions and chronological assessment of physiological and behavioral outcomes could provide insight into the functional roles associated with increased interactions. Future research should continue to explore the molecular mechanisms driving these changes and the potential for sex-specific therapeutic strategies, ultimately contributing to improved outcomes for TBI patients.

## Data Availability

The original contributions presented in the study are included in the article/supplementary material, further inquiries can be directed to the corresponding author.
